# Effect of Particulate Matter 2.5 on Primary Gingival Keratinocyte and Human Gingival Fibroblast Cell Lines

**DOI:** 10.1055/s-0044-1789269

**Published:** 2024-12-30

**Authors:** Supaporn Mala, Supranee Buranapraditkun, Kanidta Sooklert, Amornpun Sereemaspun, Puangwan Lapthanasupkul, Dulyapong Rungraungrayabkul, Nakarin Kitkumthorn

**Affiliations:** 1 Reseach Office, Faculty of Dentistry, Mahidol University, Bangkok, Thailand; 2 Division of Allergy and Clinical Immunology, Department of Medicine, King Chulalongkorn Memorial Hospital, Faculty of Medicine, Chulalongkorn University, Bangkok, Thailand; 3 Center of Excellence in Thai Pediatric Gastroenterology, Hepatology, and Immunology (TPGHAI), King Chulalongkorn Memorial Hospital, Faculty of Medicine, Chulalongkorn University, Bangkok, Thailand; 4 Center of Excellence in Nanomedicine, Department of Anatomy, Faculty of Medicine, Chulalongkorn University, Bangkok, Thailand; 5 Department of Oral and Maxillofacial Pathology, Faculty of Dentistry, Mahidol University, Bangkok, Thailand; 6 Department of Oral Medicine and Periodontology, Faculty of Dentistry, Mahidol University, Bangkok, Thailand; 7 Department of Oral Biology, Faculty of Dentistry, Mahidol University, Bangkok, Thailand

**Keywords:** PM2.5, human gingival fibroblast, primary gingival keratinocyte, cell viability, cell cycle, apoptosis

## Abstract

**Objective** Particulate matter 2.5 (PM2.5), an important air pollution particle, has been previously studied for its effects on various normal and cancer tissues. However, research on the impact of PM2.5, specifically on normal cavity tissue, is still limited. This study aimed to assess the effects of PM2.5 on cell vitality, cell cycle, and apoptosis in PGK (normal oral keratinocyte) and HGF (human gingival fibroblast) cell lines.

**Materials and Methods** The effect of PM2.5 was examined through cell vitality using the Cell Counting Kit-8 (CCK8) assay, while cell cycle and apoptosis were determined via flow cytometry. Cells incubated with 0.05% dimethyl sulfoxide were used as the negative control.

**Results** In a concentration-dependent manner, PM2.5 inhibited the proliferation of HGF and PGK cells. The half-maximal inhibitory concentration (IC50) of PM2.5 after 24 hours of incubation was 400 ng/µL for HGF cells and 100 ng/µL for PGK cells. This particulate matter arrested the cell cycles of both HGF and PGK cells at the G0/G1 phase. Additionally, PM2.5 was found to trigger apoptosis in both HGF and PGK cell lines and also cause necrosis in the PGK cell line at higher concentrations.

**Statistical Analysis** Kruskal-Wallis tests were employed to evaluate all quantitative data.

**Conclusion** The findings indicated that PM2.5 decreases cell viability, halts cell cycle progression, and triggers apoptosis in normal oral cavity cell lines. Therefore, it is advisable to avoid PM2.5 exposure in order to mitigate potential health risks. To understand PM2.5-induced oral cellular damage, more research is needed.

## Introduction

PM2.5, an acronym for particulate matter 2.5 or fine particulate matter, denotes airborne particles with a diameter of 2.5 micrometers or less. The PM2.5 chemical composition includes a mixture of different components, including organic compounds like polycyclic aromatic hydrocarbons, organic carbon, and inorganic compounds such as sulfates, nitrates, ammonium, elemental carbon (soot), and metals like lead, cadmium, and arsenic. 1

It is a prominent air pollutant, particularly concerning due to its profound respiratory system penetration. 2 3 Scientific evidence has established a connection between exposure to PM2.5 and a variety of health issues, specifically respiratory and cardiovascular conditions. 3 4 The International Agency for Research on Cancer, under the aegis of the World Health Organization, classifies outdoor air pollution, including PM2.5, as a group 1 carcinogen, reflecting its potential to cause cancer in humans. 5

The small size of PM2.5 particles enables them to deeply infiltrate the lungs and even enter the bloodstream, leading to widespread health consequences. 2 6 Additionally, an elevated risk of developing specific cancers, such as lung and bladder cancer, is associated with PM2.5 exposure. 7 8 Prolonged exposures to these fine particles can exacerbate respiratory conditions like asthma and bronchitis, impair cardiovascular function, 1 and increase the likelihood of strokes and heart attacks. 1 3

Research on the effects of PM2.5 on oral tissue in the oral cavity is not as well studied as its impact on the respiratory and cardiovascular systems. Therefore, this study aimed to investigate the impact of PM2.5 on primary gingival keratinocyte (PGK) and human gingival fibroblast (HGF) cell lines in biological terms of cell proliferation, cell cycle, and cellular apoptosis. The PGK represents oral surface epithelium, whereas HGF represents fibrous tissues.

Fibrous tissues were widely used to detect cellular responses to toxicity in the field of dentistry due to their crucial role in maintaining the structural integrity and function of oral tissues. 9 10 11 12

The study's null hypothesis stated that there was no association between PM2.5 exposure and cell apoptosis, cell cycle distribution, or cell proliferation. This work intended to shed light on these cellular reactions in order to shed further light on the potential effects of PM2.5 particles on oral health and on systemic health.

## Materials and Methods

### Chemical Agents, Cell Cultures, and Study Designs

PM2.5 was purchased from Sigma-Aldrich (St. Louis, MO). HGFs (Catalog number: 2620) isolated from human gingiva and cryopreserved at passage one was bought from ScienCell Research Laboratories, United States. These HGF cells were cultivated in Dulbecco's Modified Eagle Medium-high glucose (Gibco, United States) supplemented with 10% fetal bovine serum (Gibco, United States) under a humidified atmosphere of 5% CO _2_ at a temperature of 37°C.

The PGK cells, specifically PGKs (PCS-200-014TM), were acquired from the American Type Culture Collection. These cells were cultivated in dermal cell basal medium and supplemented with the keratinocyte growth kit. The cultivation took place in a humidified atmosphere at 37°C with 5% CO _2_ .

The PM2.5 was diluted in a solution containing 0.05% dimethyl sulfoxide (DMSO). Investigations on PM2.5 exposure were conducted using three distinct tests on HGF cells at passages 4, 5, and 6, as well as on PGK cells at passages 5, 6, and 7, to examine cell proliferation, cell cycle, and apoptosis. Every experiment was carried out three times.

### Cell Viability Assay

**Fig. 1:**
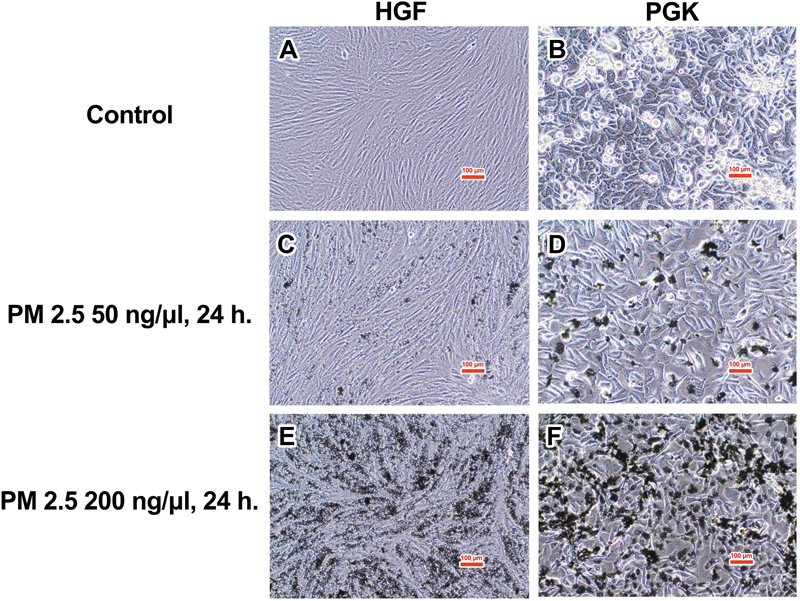
Exemplified pictures of cell lines treated with PM2.5 for 24 hours. ( **A, B** ) HGF and PGK control. ( **C, D** ) HGF and PGK treated with PM2.5 50 ng/µL. ( **E, F** ) HGF and PGK treated with PM2.5 200 ng/µL. The PM2.5 exposure investigations involved three separate tests using HGF cells (passages 4, 5, and 6) and PGK cells (passages 5, 6, and 7) to analyze cell proliferation, cell cycle, and cellular apoptosis. Each experiment was conducted three times. HGF, human gingival fibroblast; PGK, primary gingival keratinocyte.

The two cell lines were initially placed in a 96-well plate at a density of 5,000 cells per well. They were then incubated for 24, 48, and 72 hours. Following this, PM2.5 was introduced at the beginning in five different concentrations (25, 50, 100, 200, and 500 ng/µL). Examples of morphological photos captured with an inverted microscope (Olympus CKX53, Olympus Corp., Tokyo, Japan) are displayed in Fig. 1 . As a control, 0.05% DMSO was utilized. After 24, 48, and 72 hours of incubation, 10 μL of Cell Counting Kit-8 (CCK-8) reagent from Dojindo (Rightsign Trading, Bangkok, Thailand, cat no. 167008) was added to the cells for a duration of 2 hours. Next, the optical density value at a wavelength of 450 nm was measured using an Epoch 2 microtiter spectrophotometer (Agilent Technologies Inc., CA, United States). Three replicate wells were built at each time point to calculate the mean value and standard deviation (SD.).

### Cell Cycle Analysis

The impact of PM2.5 on the cell cycle was analyzed by utilizing propidium iodide (PI). Around 10,000 cells were stained with 50 µg/mL PI (Biolegend, CA, United States) in 1× phosphate-buffered saline buffer (Thermo Fisher Scientific, Inc., Waltham, MA, United States), for a duration of 30 minutes at room temperature, following a 24-hour exposure to PM2.5. Subsequently, flow cytometry was used to examine the distribution of cells with different DNA contents. The percentages of cells in sub G1, G0/G1, S, and G2/M phases were then determined using fluorescence-activated cell sorting software (BD Biosciences, San Jose, CA, United States), with an excitation wavelength of 530 nm. Three replicate wells were prepared at each time point to calculate the mean value and SD.

### Apoptosis Assay

Flow cytometry was utilized to assess the apoptosis of both cell populations by Annexin V Apoptosis Detection Kit (Biolegend, CA, United States). Around 100,000 cells were stained with fluorescein isothiocyanate (FITC) Annexin V Alexa Fluor 488 (Biolegend, CA, United States) and PI (Biolegend, CA, United States) in Annexin V Binding Buffer for 30 minutes at room temperature. Afterward, the cell types were quantified using flow cytometry (LSRII, BD Biosciences, CA, United States). The cell types and frequencies were then determined by gating based on forward scatter and side scatter. Following that, the data were analyzed using the FlowJo program (Ashland, OR, United States). On the scatter plot, the lower left quadrant represented healthy living cells (FITC −/PI − ), the lower right quadrant indicated early apoptotic cells (FITC +/PI − ), the upper right quadrant corresponded to late apoptotic cells (FITC +/PI + ), and the upper left quadrant counted as necrotic (FITC −/PI + ). The experiment was conducted three times in biological triplicate to calculate the mean and SD.

### Statistical Analysis

The mean values with SD were provided for the data, and each experiment was repeated three times. PASW Statistics (version 18.0.0) and GraphPad Prism (version 7.0) were used for statistical analysis. The Kruskal–Wallis test was employed to assess quantitative data. Statistical significance is denoted as ns (not significant), * *p* < 0.05, and ** *p* < 0.005.

## Results

### PM2.5 Inhibits Cell Proliferation of HGF and PGK Cell Lines

**Fig. 2:**
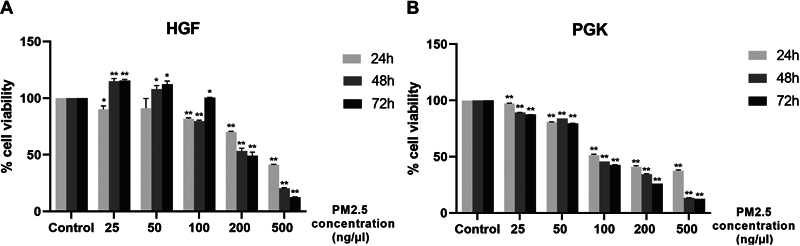
PM2.5 treatments decreased viability of ( **A** ) HGF and ( **B** ) PGK cell lines. Cells were treated with indicated concentration of PM2.5 for 24, 48 or 72 hours, and then subjected to CCK8 assay for determination of cell viability. Data were presented as the mean ± SD. Three independent experiments were performed for statistical analysis. * *p* < 0.05 and *** *p* < 0.001 as compared with dimethyl sulfoxide (DMSO) control, respectively. HGF, human gingival fibroblast; PGK, primary gingival keratinocyte; SD, standard deviation.

The viability of HGF and PGK cell lines was assessed through the CCK8 assay after exposure to different concentrations of PM2.5. Fig. 2 and Supplementary Tables S1 and S2 (available in the online version only) present all the data alongside their respective statistically significant levels. The results indicated that PM2.5 can induce cytotoxicity in a dose-dependent manner at 24, 48, and 72 hours. Specifically, at the 24-hour time point, the data showed that HGF cells exhibit concentration-dependent inhibition, with a half-maximal inhibitory concentration (IC50) value of 400 ng/µL. Interestingly, at concentrations of 25 and 50 ng/µL, the HGF cells demonstrated higher cell viability after 48 and 72 hours of incubation. At a concentration of 100 ng/µL, the HGF cells showed improved vitality after 72 hours. This observation suggested that HGF cells possess a certain level of tolerance when exposed to low concentrations of PM2.5. On the other hand, the IC50 for PGK cells was 100 ng/µL after 24 hours. Compared to HGF cells, PGK cells exhibited a higher susceptibility to PM2.5, as demonstrated by a decline in the number of viable cells at each PM2.5 concentration over time. Hence, it is possible to draw the conclusion that PM2.5 possesses the higher capacity to impede the proliferation of cells that are not malignant, particularly epithelial cells.

### PM2.5 Induces Cell Cycle Arrest at the G0/G1 Phase

**Fig. 3:**
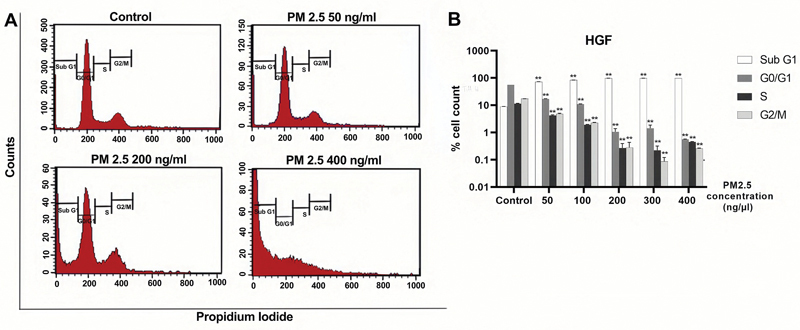
PM2.5 treatments induced G0/G1 arrest of HGF cells. Representative cell cycle distribution of HGF cells exposed to a serial concentration of PM2.5 for 24 hours was shown. ( **A** ) Cell cycle gating. ( **B** ) Histogram. Percentages of different cell cycle phases, including sub G1, G0/G1, S, and G2/M, were presented. Data were presented as the mean ± SD. Three independent experiments were performed for statistical analysis. * *p* < 0.05 and ** *p* < 0.001 as compared with the same cell cycle phase of dimethyl sulfoxide (DMSO) control, respectively. HGF, human gingival fibroblast; SD, standard deviation.

**Fig. 4:**
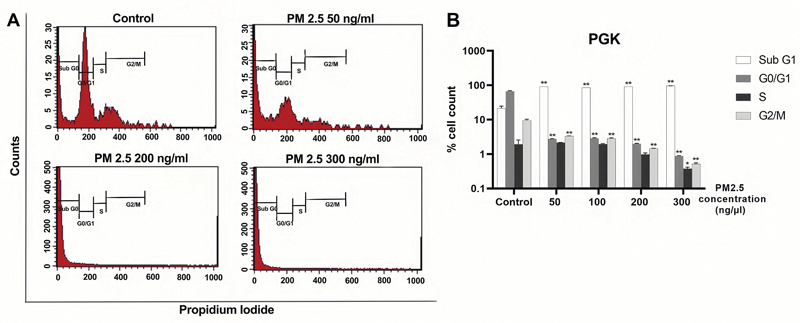
PM2.5 treatments induced G0/G1 arrest of PGK cells. Representative cell cycle distribution of PGK cells exposed to a serial concentration of PM2.5 for 24 hours was shown. ( **A** ) Cell cycle gating. ( **B** ) Histogram. Percentage of different cell cycle phases, including sub G1, G0/G1, S, and G2/M, were presented. Data were presented as the mean ± SD. Three independent experiments were performed for statistical analysis. * *p* < 0.05 and ** *p* < 0.001 as compared with the same cell cycle phase of dimethyl sulfoxide (DMSO) control, respectively. PGK, primary gingival keratinocyte; SD, standard deviation.

Flow cytometric analysis was conducted to examine the distribution of the cell cycle in both cell lines after 24 hours of PM2.5 treatment. Figs. 3 and 4 display the results and statistically significant levels, while Supplementary Tables S3 and S4 (available in the online version only) provide detailed information. These data illustrated the percentages of HGF and PGK cell lines during each stage of the cell cycle following PM2.5 treatment. We observed the effect of PM2.5 in both cell lines, resulting in their arrest at the G0/G1 phase. This arrest was observable when the cells were treated with PM2.5 at a concentration of 50 ng/µL. The results showed a notable increase in the proportion of cells in the sub G1 phase, accompanied by a decrease in the percentages of cells in the G0/G1, G2/M, and S phases of the cell cycle in both cell lines. These findings strongly suggest that PM2.5 hinders the proliferation of both cell lines and impedes their progression to the G0/G1 phase.

### PM2.5 Enhances Apoptotic Effect on PGK and HGF Cell Lines

**Fig. 5:**
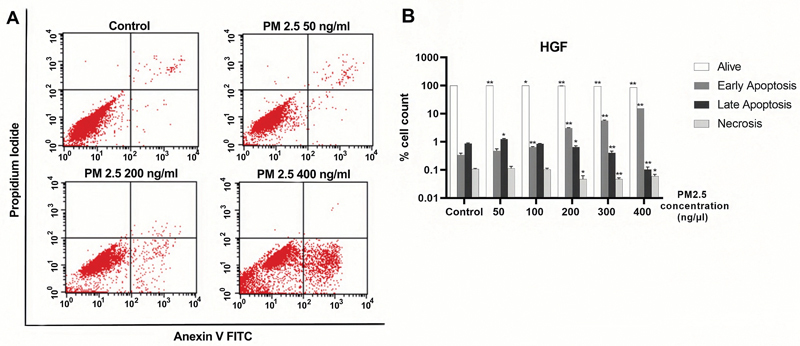
PM2.5 treatments induced early apoptosis of HGF cells. HGF cells were exposed to a serial concentration of PM2.5 for 24 hours. ( **A** ) Flow cytometry gating. ( **B** ) Histogram. Percentage of alive, early apoptosis, late apoptosis, and necrosis were presented. Data were presented as the mean ± SD. Three independent experiments were performed for statistical analysis. * *p* < 0.05 and ** *p* < 0.001 as compared with dimethyl sulfoxide (DMSO) control, respectively. HGF, human gingival fibroblast; SD, standard deviation.

**Fig. 6:**
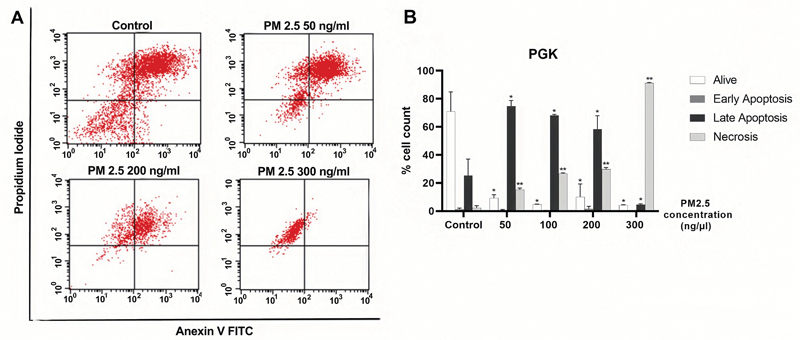
PM2.5 treatments induced late apoptosis and necrosis cells of PGK cells. PGK cells were exposed to a serial concentration of PM2.5 for 24 hours. ( **A** ) Flow cytometry gating. ( **B** ) Histogram. Percentage of alive, early apoptosis, late apoptosis, and necrosis were presented. Data were presented as the mean ± SD. Three independent experiments were performed for statistical analysis. * *p* < 0.05 and ** *p* < 0.001 as compared with dimethyl sulfoxide (DMSO) control, respectively. PGK, primary gingival keratinocyte; SD, standard deviation.

We have confirmed that PM2.5 triggers apoptosis, as demonstrated by the increased exposure of phosphatidylserine. Thereafter, we conducted flow cytometry analysis to examine the expression of annexin V-FITC/PI. The results of flow cytometry analysis were gated and are presented in Figs. 5A and 6A . According to the results obtained from the annexin V-FITC/PI staining, we noticed a significant increase in the rate of early apoptosis in HGF cells exposed to PM2.5 in a dose-dependent manner. In contrast, within the PGK cell line, the rate of late apoptosis was found to be highest at 50 ng/µL compared to the control group, followed by a gradual decrease at 100, 200, and 300 ng/µL. Notably, we observed an increase in necrosis in PGK cell lines in a concentration-dependent manner. Specifically, at a dose of 300 ng/µL, PM2.5 induced necrosis in 91% of the PGK cells. These data indicated a significant impact on necrosis from high-dose PM2.5. The corresponding data with statistically significant levels are presented in Figs. 5B and 6B , while more detailed information on the apoptosis data is available in Supplementary Tables S5 and S6 (available in the online version only).

## Discussion

The findings of this study demonstrated concentration-dependent, statistically significant ( *p* < 0.05) associations between PM2.5 levels and decreased cell viability, G0/G1 cell cycle arrest, and elevated cellular apoptosis. As a result, the null hypothesis was rejected.

The PM2.5 particles are tiny, can easily be inhaled deep into the lungs, and may even enter the bloodstream. This can lead to oxidative stress and inflammation in the body, as these particles contain harmful substances such as heavy metals, inorganic elements, organic matter, etc. 1 13 Chronic exposure to PM2.5 has been associated to various health issues, including respiratory and cardiovascular disease. 3 It is important to highlight that while PM2.5 is a concern, it is not the sole cause of cancer. In normal bronchial epithelium, it can stimulate oxidative stress, apoptosis, and inflammatory responses. 14 Inhalation of PM2.5 can lead to irritation and inflammation in the respiratory tract and passages, including the oral mucosa. 15 This irritation can cause discomfort and may contribute to oral health issues that may increase the risk of tooth decay, periodontal disease, potentially malignant oral disorders, and oral cancer. 15

Our research focused on the impact of PM2.5 on the growth inhibition, cell cycle arrest, and apoptosis in HGF and PGK cells. These two cell lines exhibit distinct cell types, with HGF being a fibroblast representing the underlying connective tissue, while PGK is an epithelial cell representing the superficial epithelial layer. The PGK cell line exhibited a higher sensitivity to low concentrations of PM2.5 when compared to the HGF cell line. Furthermore, the PGK exhibited a significant increase in late apoptosis rate, while the HGF showed a higher percentage of early apoptotic cells due to the increase in PM2.5 levels. The varying characteristics of these cell lines may explain the differences observed. Our results suggest that the connective tissue, as observed in HGF, may have an over-resistance to PM2.5 above the epithelium.

PM2.5 has been shown to induce proliferation inhibition through various pathways, including cell cycle arrest and cell death. In our study, PM2.5 led to cell cycle arrest at the G0/G1 phase in both HGF and PGK cells. This is consistent with previous findings that demonstrated G0/G1 cell cycle arrest in human HaCaT keratinocytes following PM2.5 exposure. 16 Additionally, after 24 and 48 hours of PM2.5 treatment, there was an increase in cell number at the sub G1 phase accompanied by a decrease at the G0/G1 phase, indicating a reduction in cell viability over time. This reduction in cell viability suggests a decreased rate of DNA synthesis after PM2.5 treatment. The transition from a dormant quiescent stage (G0) to an actively growing state is crucial for cell cycle entry and proliferation in most cells.

Cell cycle progression is regulated by various cyclin-dependent kinase (CDK)–cyclin complexes. 17 A prior investigation demonstrated that PM2.5 downregulates the expression of key cell cycle regulatory proteins associated with G0/G1-phase progression, including cyclin D1, cyclin E, Cdk2, and Cdk4. 16 Additionally, research by Zhang et al indicated that PM2.5 induces cell cycle arrest and suppresses cyclin D1 expression via the mTOR/P70S6K1 signaling pathway. 18 Moreover, Herath et al observed an upregulation of the Cdk inhibitory proteins, p21 and p27, in response to PM2.5. 16 Nevertheless, further studies are warranted to elucidate the underlying mechanisms of PM2.5-induced cell cycle alterations in both associated proteins and pathways.

PM2.5 has been implicated in various modes of cell death, including autophagy, apoptosis, ferroptosis, pyroptosis, and necrosis. 19 20 Specifically, PM2.5 has been shown to induce autophagy and apoptosis through endoplasmic reticulum stress in human endothelial cells. 21 Consistent with G0/G1 phase arrest, Yuan et al demonstrated that PM2.5 induces apoptosis by altering the Bcl-2/Bax protein ratio and modulating the transcription levels of p15 ^INK4B^ , p16 ^INK4A^ , and p21 ^WAF1/CIP1^ . 22 In addition, PM2.5 enhances sensitivity to ferroptosis, which is characterized by increased ferroptotic events in endothelial cells, where iron overload, lipid peroxidation, and redox imbalance play pivotal roles. 23 PM2.5 has also been implicated in inducing pyroptosis through the NLRP3 inflammasome pathway. 24 Furthermore, our study revealed that high doses of PM2.5 cause cell necrosis, consistent with observations from previous research. 25 Taken together, further studies are needed to fully grasp the fundamental processes at play, particularly the involvement of cell death proteins. Taken together, further studies are needed to fully understand the underlying processes, particularly the types of cell death and the role of cell death-related proteins.

There are many cell viability assays that can be used. For instance, a trypan blue exclusion assay is a qualitative method that solely detects the viability of a cell. 26 The MTT (3-(4,5-dimethylthiazol-2-yl)-2,5-diphenyltetrazolium bromide) assay is commonly employed to measure reductive metabolism in cells for assessing viability, proliferation, and cytotoxicity. 27 However, the CCK-8 test was selected to evaluate cell viability in this investigation. The CCK-8 assay has higher sensitivity compared to the MTT assay as it involves a larger proportion of the dehydrogenase enzymes present in a cell, whereas the MTT assay solely involves mitochondrial dehydrogenase. Hence, the MTT assay relies on the functionality of mitochondria rather than the overall cell. 28

One limitation of this work was using Annexin V-FITC/PI for identifying apoptosis and necrosis. PI, a nucleic acid stain, only penetrates dead cells, making it unsuitable for viable cells. While it helps distinguish live, apoptotic, and necrotic cells when used with Annexin V, its inability to stain viable cells can affect the precision in differentiating early apoptotic cells from late apoptotic or necrotic ones. 29 Further study on another technique, known as 7AAD (7-aminoactinomycin D), which offers an advantage over PI, should be considered. 7AAD is more precise in distinguishing between early apoptotic cells, which still have intact membranes, and late apoptotic/dead cells, where membrane integrity has been damaged. 30 Another main limitation was that it solely relied on *in vitro* experiments, which were performed in controlled laboratory conditions on two specific cell lines (PGK and HGF). Thus, the findings should be considered initial evidence, emphasizing the significance of being careful with PM2.5 that comes into contact with oral tissues. This also highlights the need for additional data in this matter. In order to accurately assess the clinical effectiveness of PM2.5 on normal oral cells, further *in vitro* and *in vivo* studies on additional normal oral cell lines are necessary. Moreover, there is a requirement for an in-depth exploration of the exact mechanism underlying PM2.5's antiproliferative and apoptotic effects.

PM2.5 is currently an important issue in the context of global warming. 31 To protect against the adverse effects of PM2.5, it is critical to support efforts to improve air quality and reduce exposure to air pollution. This objective can be accomplished by implementing regulatory actions, embracing cleaner energy alternatives, advocating for public transportation, and employing personal protective measures like donning masks during periods of poor air quality. 32 Such comprehensive approaches are vital for safeguarding public health and diminishing the adverse effects associated with PM2.5 pollution.

## Conclusion

The present study serves as the starting point for future investigation of PM2.5 reduced cell vitality in normal oral cavity cells, which is contributed by cell cycle arrest and apoptosis. To our knowledge, this is the first to demonstrate the antiproliferative and apoptosis-inducing effects of PM2.5 in HGF and PGK cells. Hence, its potential to cause oral cavity tissue damage rather than lung tissue is novel. However, further evidences are required to draw conclusions from the observed results.
