# Supplementary material for: Effect of Particulate Matter 2.5 on Primary Gingival Keratinocyte and Human Gingival Fibroblast Cell Lines
Source: Eur J Dent. 2024 Dec 30;19(2):464–71. doi: 10.1055/s-0044-1789269 (PMC12020600; doi:10.1055/s-0044-1789269)
Supplement: Supplementary file 1 — Supplementary Material [file 10-1055-s-0044-1789269-s2443472.pdf]

Supplementary Table S1 Percentage of HGF cell viability after treatment with PM2.5 (mean ± SD)

PM 2.5 concentration	24 h	48 h	72 h
25 ng/µL	90.22 ± 2.94	114.92 ± 2.42	115.38 ± 1.18
50 ng/µL	91.19 ± 8.50	108.11 ± 2.96	112.29 ± 2.80
100 ng/µL	81.80 ± 0.85	79.79 ± 0.71	100.49 ± 0.24
200 ng/µL	70.15 ± 0.49	53.44 ± 2.35	49.23 ± 3.09
500 ng/µL	41.31 ± 0.27	20.44 ± 0.48	12.57 ± 0.25

Abbreviations: HGF, human gingival fibroblast; SD, standard deviation.

Supplementary Table S2 Percentage of PGK cell viability after treatment with PM2.5 (mean ± SD)

PM 2.5 concentration	24 h	48 h	72 h
25 ng/µL	97.26 ± 0.29	89.30 ± 0.05	87.43 ± 0.07
50 ng/µL	80.82 ± 0.17	83.95 ± 0.04	79.61 ± 0.05
100 ng/µL	51.60 ± 0.61	45.82 ± 0.03	42.71 ± 0.08
200 ng/µL	41.13 ± 0.97	34.45 ± 0.49	25.99 ± 0.01
500 ng/µL	37.44 ± 0.70	13.38 ± 0.03	12.57 ± 0.03

Abbreviations: PGK, primary gingival keratinocyte; SD, standard deviation.

Supplementary Table S3 Percentage of HGF cell cycles after treatment with PM2.5 for 24 hours (mean ± SD)

PM 2.5 concentration	Sub G1	G0/G1	S	G2/M
Control	8.92 ± 0.09	56.37 ± 0.07	11.50 ± 0.29	17.32 ± 0.10
50 ng/µL	72.67 ± 0.31	17.21 ± 0.21	4.24 ± 0.20	4.80 ± 0.30
100 ng/µL	84.20 ± 0.11	11.11 ± 0.18	1.94 ± 0.04	2.29 ± 0.07
200 ng/µL	98.36 ± 0.63	1.06 ± 0.33	0.27 ± 0.12	0.28 ± 0.15
300 ng/µL	98.25 ± 0.56	1.45 ± 0.43	0.22 ± 0.10	0.09 ± 0.04
400 ng/µL	98.64 ± 0.03	0.57 ± 0.01	0.45 ± 0.01	0.26 ± 0.01

Abbreviations: HGF, human gingival fibroblast; SD, standard deviation.

Supplementary Table S4 Percentage of PGK cell cycles after treatment with PM2.5 for 24 hours (mean ± SD)

PM 2.5 concentration	Sub G1	G0/G1	S	G2/M
DMSO	21.56 ± 3.18	66.74 ± 2.56	1.93 ± 0.63	9.50 ± 0.80
50 ng/µL	91.60 ± 0.11	2.79 ± 0.02	2.17 ± 0.02	3.35 ± 0.03
100 ng/µL	86.15 ± 0.11	2.89 ± 0.09	1.94 ± 0.07	2.82 ± 0.14
200 ng/µL	90.70 ± 0.18	2.01 ± 0.04	0.98 ± 0.10	1.45 ± 0.03
300 ng/µL	96.24 ± 0.09	0.88 ± 0.02	0.38 ± 0.04	0.52 ± 0.04

Abbreviations: PGK, primary gingival keratinocyte; SD, standard deviation.

 © 2024. The Author(s).

Supplementary Table S5 Percentage of HGF cell apoptosis and necrosis after treatment with PM2.5 for 24 hours (mean ± SD)

PM2.5 concentration	Alive	Early apoptosis	Late apoptosis	Necrosis
DMSO	98.68 ± 0.07	0.34 ± 0.05	0.87 ± 0.03	0.11 ± 0.01
50 ng/μL	98.17 ± 0.05	0.48 ± 0.08	1.24 ± 0.07	0.11 ± 0.02
100 ng/μL	98.41 ± 0.03	0.64 ± 0.03	0.84 ± 0.03	0.10 ± 0.01
200 ng/μL	96.24 ± 0.16	3.07 ± 0.08	0.65 ± 0.07	0.05 ± 0.02
300 ng/μL	93.89 ± 0.30	5.65 ± 0.25	0.40 ± 0.06	0.05 ± 0.01
400 ng/μL	84.38 ± 0.04	15.46 ± 0.05	0.10 ± 0.02	0.06 ± 0.01

Abbreviations: HGF, human gingival fibroblast; SD, standard deviation.

Supplementary Table S6 Percentage of PGK cell apoptosis and necrosis after treatment with PM2.5 for 24 hours (mean ± SD)

PM2.5 concentration	Alive	Early apoptosis	Late apoptosis	Necrosis
DMSO	71.08 ± 13.81	1.26 ± 0.83	25.40 ± 11.65	2.27 ± 1.56
50 ng/μL	9.20 ± 2.46	0.71 ± 0.40	74.78 ± 3.98	15.31 ± 1.11
100 ng/μL	4.67 ± 0.28	0.43 ± 0.10	68.21 ± 0.67	26.69 ± 0.48
200 ng/μL	10.16 ± 9.16	1.67 ± 1.68	58.41 ± 9.56	29.77 ± 1.29
300 ng/μL	4.17 ± 0.30	0.00 ± 0.00	4.62 ± 0.53	91.21 ± 0.43

Abbreviations: PGK, primary gingival keratinocyte; SD, standard deviation.

 © 2024. The Author(s).
